# Dominant Suppression of Inflammation via Targeted Mutation of the mRNA Destabilizing Protein Tristetraprolin

**DOI:** 10.4049/jimmunol.1402826

**Published:** 2015-05-22

**Authors:** Ewan A. Ross, Tim Smallie, Qize Ding, John D. O’Neil, Helen E. Cunliffe, Tina Tang, Dalya R. Rosner, Iva Klevernic, Nicholas A. Morrice, Claudia Monaco, Adam F. Cunningham, Christopher D. Buckley, Jeremy Saklatvala, Jonathan L. Dean, Andrew R. Clark

**Affiliations:** * School of Immunity and Infection, College of Medical and Dental Sciences, University of Birmingham, Birmingham B15 2TT, United Kingdom;; † Imperial College London, Hammersmith Hospital, London W12 0NN, United Kingdom;; ‡ Unit of Signal Transduction, Interdisciplinary Cluster for Applied Genoproteomics, University of Liege, University Hospital, 4000 Liege, Belgium;; § Beatson Institute for Cancer Research, Bearsden, Glasgow G61 1BD, United Kingdom; and; ¶ Kennedy Institute of Rheumatology, Nuffield Department of Orthopaedics, Rheumatology and Musculoskeletal Sciences, University of Oxford, Oxford OX3 7FY, United Kingdom

## Abstract

In myeloid cells, the mRNA-destabilizing protein tristetraprolin (TTP) is induced and extensively phosphorylated in response to LPS. To investigate the role of two specific phosphorylations, at serines 52 and 178, we created a mouse strain in which those residues were replaced by nonphosphorylatable alanine residues. The mutant form of TTP was constitutively degraded by the proteasome and therefore expressed at low levels, yet it functioned as a potent mRNA destabilizing factor and inhibitor of the expression of many inflammatory mediators. Mice expressing only the mutant form of TTP were healthy and fertile, and their systemic inflammatory responses to LPS were strongly attenuated. Adaptive immune responses and protection against infection by *Salmonella typhimurium* were spared. A single allele encoding the mutant form of TTP was sufficient for enhanced mRNA degradation and underexpression of inflammatory mediators. Therefore, the equilibrium between unphosphorylated and phosphorylated TTP is a critical determinant of the inflammatory response, and manipulation of this equilibrium may be a means of treating inflammatory pathologies.

## Introduction

The finely orchestrated programs of gene expression in immune cells responding to stimulation are dictated not only by transcriptional regulation but equally by posttranscriptional processes, in particular the control of mRNA stability (1–4). Many cytokines, chemokines, and other immune mediators are encoded by mRNAs that have intrinsically short half-lives, with their rapid turnover being essential for timely termination of immune responses. Furthermore, the modulation of rates of mRNA destruction by pro- and anti-inflammatory agonists is an important means of controlling the duration and quality of those responses. For example, the p38 MAPK signaling pathway is activated by proinflammatory stimuli and mediates transient stabilization of many inflammatory mediator mRNAs (reviewed in Ref. 5). The aberrant expression of proinflammatory mediators in pathological conditions cannot be fully understood without investigating posttranscriptional mechanisms.

Tristetraprolin (TTP) is the founding member of a small family of evolutionarily conserved, sequence-specific RNA binding proteins, which is encoded by the *Zfp36* gene in the mouse and recognizes the optimum binding site WUAUUUAUW (where W is adenosine or uridine) (6). TTP binds to this sequence element in the 3′ untranslated region (UTR) of target transcripts, including *Tnf* and many other inflammatory factors. It then mediates recruitment of the carbon catabolite repression protein 4/carbon catabolite repression protein 4–associated factor 1 deadenylase complex and thus promotes the shortening of the poly(A) tail of the target mRNA (7–11). In most cases, this is rapidly followed by the destruction of the mRNA body (12). Hence, TTP is a critical negative regulator of expression of a large number of proinflammatory genes (6). *Zfp36^−/−^* mice lacking TTP protein have a severe, pervasive inflammatory phenotype that includes cachexia, dermatitis, autoimmunity, and inflammatory arthritis. The phenotype is largely (although not exclusively) due to increased stability of *Tnf* mRNA leading to increased expression of TNF protein in the myeloid compartment (3, 6, 13).

A working model of the posttranscriptional regulation of proinflammatory gene expression by the p38 MAPK pathway has been built up using a variety of in vitro assays, transient transfections of reporter construct, and studies of endogenous transcripts in cells derived from knockout mice (reviewed in Refs. 14, 15). According to this model, p38 MAPK activates the downstream kinase MAPK-activated protein kinase 2 (MK2), which phosphorylates TTP at serines 52 and 178 (murine TTP residue numbers), preventing the recruitment of the carbon catabolite repression protein 4/carbon catabolite repression protein 4–associated factor 1 complex and hence inhibiting deadenylation and promoting stabilization of TTP target transcripts. TTP is not expressed by resting macrophages, and for a number of reasons its accumulation depends on the p38 MAPK pathway. First, transcription of the *Zfp36* gene is controlled by MK2 (15). Second, p38 MAPK and MK2 regulate the stability of *Zfp36* mRNA via an autoregulatory loop in which TTP recognizes a binding site in its own (i.e., *Zfp36*) 3′ UTR (3, 16). Third, the same MK2-mediated phosphorylations that cause inactivation of TTP protect it from degradation by the proteasome (17, 18).

We and others have hypothesized that the complex, multilayered regulation of TTP expression and function permits tight coupling between the activity of the p38 MAPK pathway and the expression of proinflammatory genes (3, 17, 18). In the early phase of an inflammatory response, in which p38 MAPK activity is typically high, TTP protein accumulates in a dormant form as the phosphorylation of serines 52 and 178 promotes protein stabilization and inactivation. This permits the accumulation and translation of TTP target transcripts that encode mediators of inflammation. When p38 MAPK activity subsequently falls, TTP is activated via dephosphorylation, likely by PP2A (19), and drives the off-phase of the inflammatory response by promoting the rapid deadenylation of target transcripts. In vitro assays and overexpression experiments provide circumstantial evidence in support of this hypothesis. The experiments described in this study were intended to test in vivo consequences of perturbing the MAPK p38-mediated control of TTP function and to explore the concept of therapeutic targeting of the equilibrium between phosphorylated and unphosphorylated TTP.

## Materials and Methods

### Reagents

LPS (*Escherichia coli* serotype EH100) was purchased from Enzo Life Sciences. Other biochemicals were purchased from Sigma-Aldrich unless otherwise stated. All media and sera were routinely tested for endotoxin using the *Limulus* amebocyte lysate test (Lonza) and were rejected when the endotoxin concentration was >0.1 U/μl.

### Generation of a cell line stably expressing flag-TTP

RAW-MB01, a RAW264.7 clone expressing a tetracycline-responsive (Tet-Off) transcription factor, was generated by stable transfection and selection using blasticidin (2 μg/ml). LPS-induced expression of *Tnf* mRNA and TNF protein in this clone was indistinguishable from that in the parental RAW264.7 line (data not shown). Murine TTP cDNA with an N-terminal flag tag was subcloned into the tetracycline-responsive expression vector pTRE2Hyg (TaKaRa Bio). The vector was transfected into RAW-MB01, and several clones resistant to hygromycin (100 μg/ml) were isolated. Clone RAW-MB01-WT3.1 was used in the present study. Expression of flag-TTP was found to be strongly inducible by LPS but only weakly responsive to tetracycline, because of leakiness of the tetracycline-regulated promoter in RAW264.7 cells (data not shown).

### Phosphoproteomics

RAW-MB01-WT3.1 cells were stimulated for 2 h with 10 ng/ml LPS and lysed in lysis buffer (50 mM Tris-HCl [pH 7.5], 1 mM EDTA, 1 mM EGTA, 1 mM Na_3_VO_4_, 10 mM sodium β-glycerophosphate, 5 mM sodium pyrophosphate, 50 mM NaF, 0.27 M sucrose, 1% [w/v] Nonidet P-40, 1 mM DTT, 1 μg/ml pepstatin, 1 mM PMSF, 10 mM E64). All purification steps were at 4°C. Lysates were precleared overnight using protein G–agarose beads and then applied to M2 anti-flag agarose beads for 4 h. Beads were washed six times with LB plus 0.25 M NaCl and three times with LB plus 0.15 M NaCl, and then flag-TTP was eluted in EB (50 mM Tris-HCl [pH 7.5], 150 mM NaCl, 0.1 mM EGTA) containing 0.25 mg/ml 3× flag peptide. The eluate was subjected to SDS-PAGE, bands were visualized using colloidal Coomassie blue, excised, digested with trypsin, and analyzed by liquid chromatography–mass spectrometry on an LTQ-Orbitrap-XL essentially as described previously. Peptide mixtures were separated on a 150 × 0.075 mm PepMapC18 column fitted to a Proxeon Easy-LC and liquid chromatography–tandem mass spectrometry was performed using multistage activation in the LTQ. Raw data files were searched against Swiss-Prot database (160613) using Mascot 2.4 (Matrix Science) and processed using Proteome Discoverer 1.3 software (Thermo Scientific). Search criteria were: precursor mass tolerance, 20 ppm; tandem mass spectrometry mass tolerance, 0.6 Da; enzyme, trypsin (KP/RP bonds cleaved, two missed cleavages permitted); variable modifications, phosphorylation (STY) and oxidation (M); minimum peptide ion score, 20. Extracted ion chromatography was performed using Proteome Discoverer 1.3 and Xcalibur software (Thermo Scientific).

### Generation and testing of phospho-specific antisera

An antiserum specific for phosphorylated Ser^52^ was generated by immunization of rabbits with the peptide LTGRSTpSLVEGR (Eurogentec). The serum was precleared using the equivalent nonphosphorylated peptide and then affinity purified against the phosphopeptide. An antiserum specific for phosphorylated Ser^178^ was generated by immunization of rabbits with the peptide LRQSIpSFSGLPSR (Division of Signal Transduction Therapy, University of Dundee). The serum was affinity purified using the immunizing peptide. Codons 52 and 178 in pGEX-TTP (20) were mutated from serine to alanine codons using QuikChange (Agilent Technologies). Wild-type GST-TTP and GST-TTP-S52A/S178A were purified as described (20). In vitro phosphorylations were performed at 30°C for 30 min with 3 μg recombinant TTP substrate and 100 μM ATP in the absence or presence of 0.01 U purified MK2 (Millipore). Aliquots containing 100 ng TTP were transferred to nitrocellulose and blotted with either phospho-specific antisera or antiserum raised against total TTP (20).

### Generation of *Zfp36aa/aa* strain

Targeting of the *Zfp36* was performed with the assistance of Genoway (see schematic in Fig. 2A). Homology arms were generated by PCR from 129Sv genomic DNA and sequenced in their entirety. The targeting construct was created by sequential ligation of the two homology arms, a neomycin cassette flanked by Cre recombinase recognition sites and a synthetic DNA fragment (Top Gene Technologies) corresponding to part of the *Zfp36* intron and part of the second exon, incorporating serine to alanine substitutions at codons 52 and 178. The construct encopasses a 9.5-kb region orthologous to Chr7:28,372,130–28,381,646 of the C57BL/6J genome (build 38). The position of insertion of the neomycin cassette corresponds to Chr7:28,378,880. The targeting construct was transfected into 129Sv embryonic stem cells. Correct recombination of the *Zfp36* locus was confirmed by Southern blotting, PCR, and sequencing of genomic DNA for two independent neomycin-resistant embryonic stem cell clones. Recombinant embryonic stem cells were injected into C57BL/6J blastocysts, which were then reimplanted into pseudo-pregnant C57BL/6J females. Three males with >50% coat color chimerism were selected for breeding with C57BL/6J female mice constitutively expressing Cre recombinase (Cre deleter strain). Twenty-three of 23 pups had Agouti coat color, suggesting germline transmission of the modified *Zfp36* allele. In three of those pups, correct excision of the neomycin cassette was confirmed by Southern blotting, genomic DNA sequencing, and PCR of genomic DNA with primers flanking the site of the neomycin insert: CLA1 forward (5′-CACACCTGTCTAGTGCCCTTCGCTG-3′) and CLA1 reverse (5′-CCCAAATCAAAGCACACTGCTGCTC-3′). Two *Zfp36^+^/aa* males were used as founders of a breeding colony, with genotyping using the above oligonucleoties. Initial experiments used littermate *Zfp36^+/+^* and *Zfp36aa/aa* mice. The strain has now been back-crossed against C57BL/6J for 10 generations. Parallel, true breeding *Zfp36^+/+^* and *Zfp36aa/aa* lines were then established from progeny of *Zfp36^+^/aa* versus *Zfp36^+^/aa* matings, and were used in all experiments described in the present study. Differences between *Zfp36^+/+^* and *Zfp36aa/aa* mice were consistent whether littermates or true-breeding *Zfp36^+/+^* and *Zfp36aa/aa* lines were used.

### In vivo experiments and cell isolation

All animal experiments were approved by local ethical committees and performed under UK Home Office project licenses. C57BL/6 mice were purchased from Harlan Laboratories UK. All mice used were between 6 and 12 wk of age. To assess the response to LPS-induced systemic shock, mice were injected i.p. with 5 mg/kg purified LPS (Serotype EH100, Enzo Life Sciences) in 200 μl sterile PBS. Mice were culled 3 or 12 h after challenge, peripheral blood was collected by cardiac puncture for serum isolation, and tissues were frozen in liquid nitrogen for histology. Serum markers of tissue damage were measured by the Clinical Pathology Service Laboratory at MRC Harwell (Didcot, U.K.). The response to bacterial infection was assessed by challenging mice with 5 × 10^5^ attenuated *Salmonella enterica* serovar *typhimurium* as previously described (21).

Bone marrow–derived macrophages (BMMs) were generated from hindlegs of humanely culled animals and differentiated in vitro with 100 ng/ml M-CSF (PeproTech) in 10% heat-inactivated FCS RPMI 1640 containing penicillin/streptomycin for 7 d. BMMs were plated at a density of 1 × 10^6^/ml in the appropriate cell culture plate at least 1 d prior to stimulation. Primary peritoneal macrophages were harvested by lavaging the peritoneal cavity with 5 ml PBS containing 2 mM EDTA. Lavage fluid was collected and cells were resuspended at 2 × 10^6^/ml in DMEM supplemented with 10% heat-inactivated FCS and penicillin/streptomycin. Cells were plated and allowed to adhere for 1 h at 37°C, before being washed twice with media. The remaining adherent cells were >90% F4/80^+^ macrophages as assessed by flow cytometry. Cells were rested overnight before stimulation. Mesenteric and inguinal lymph node T cells were negatively selected using Pan T Cell Isolation Kit II (Miltenyi Biotec) and stimulated for 72 h with plate-bound anti-CD3ε and anti-CD28 (eBioscience). Spleen cells were harvested as described (21) and stimulated for 6 h with plate-bound anti-CD3ε and anti-CD28, the last 3 h in the presence of brefeldin A (3 μg/ml). Intracellular IFN-γ was measured as described (22), using a Cyan ADP flow cytometer (Dako) and FloJo software (Tree Star).

### Measurement of mRNA

RNA was extracted from BMMs using QIAshredder columns and RNeasy Mini kits (Qiagen). cDNA was generated using iScript cDNA synthesis kit (Bio-Rad). Gene expression was quantified by real-time PCR on a LightCycler 480 II (Roche) using SuperScript III platinum RT-PCR kit and custom-synthesized oligonucleotide primers (Eurofins MWG) with SYBR Premix Ex Taq (Lonza). Relative gene expression was calculated using the ΔΔCt method with *Gapdh* mRNA for normalization of RNA levels. Sequences of oligonucleotides are available upon request from the authors.

For microarray analysis, RNA was extracted as above and purified using RNA Clean and Concentrator kits (Cambridge Bioscience). Hybridization and analysis were performed by Oxford Gene Technology. Total RNA or control RNA were converted to labeled cRNA with Cy3 or Cy5, respectively, using a two-color low input Quick Amp labeling kit (Agilent Technologies). Samples were hybridized onto SurePrint G3 mouse GE 8 × 60k slides and read using a G2505C scanner (Agilent Technologies). The scanned images were analyzed with Agilent Feature Extraction software 10.7.3.1 using default parameters. Processed signal intensities were background subtracted and spatially detrended.

### RNA immunoprecipitation

BMMs (∼2 × 10^7^) were left untreated or stimulated with 10 ng/ml LPS for 1 h, harvested, and washed twice with ice-cold PBS, then lysed in 1 ml ice-cold polysome lysis buffer (100 mM KCl, 10 mM HEPES [pH 7.0], 5 mM MgCl_2_, 0.5% Nonidet P-40, 1 mM DTT, 100 U/ml RNase inhibitor, protease inhibitor mixture [Roche]). Lysates were frozen at −80°C, thawed, and cleared by centrifugation in a chilled benchtop centrifuge at 13,000 rpm for 5 min. Protein A beads were washed three times in NT2 buffer (150 mM NaCl, 50 mM HEPES [pH 7.4], 1 mM MgCl_2_, 5% BSA) and then incubated overnight with TTP-specific antiserum or rabbit IgG. Beads were washed four times with buffer NT2 minus BSA, plus 1 mM DTT, 15 mM EDTA, 100 U/ml RNase inhibitor, and protease inhibitors. Beads were resuspended in the same modified NT2 buffer, added to macrophage lysates, and incubated overnight. Beads were washed four times in buffer NT2, and RNA was extracted using TRIzol reagent according to the manufacturer’s instructions. An equal volume of isopropanol and 20 μg glycogen were added, and RNA was precipitated by incubation at −20°C for 1 h and centrifugation at 13,000 rpm for 5 min at 4°C. The pellet was washed with 70% ethanol, resuspended in distilled water, and used as a template for RT-PCR as described above.

### Assessment of protein expression

Secreted factors in tissue culture supernatants and sera were quantified by ELISA according to the manufacturer’s instructions (eBioscience), or by using mulitplex bead capture assays and a Bio-Plex 200 analyzer (Bio-Rad). Cell lysates were resolved on SDS-PAGE gels, probed with primary Abs, and immunoreactive proteins were visualized with HRP-coupled secondary Abs and chemiluminescence reagents (Bio-Rad, Pierce, or Cell Signaling Technology). Blots were visualized using the ChemiDoc MP imaging system and in some cases were quantified using Image Lab software (Bio-Rad).

### Adenoviral reporters

The construction of adenoviral reporter constructs containing the human TNF 5′ region, the TNF 5′ region, and 3′ UTR, or five tandem copies of an NF-κB consensus was previously described, as was the method of macrophage infection (23). Levels of viral infection were quantified by flow cytometric assessment of GFP fluorescence. Luciferase assays were performed using the Bright-Glo luciferase assay system (Promega). Luminescence was measured with a Centro LB 960 luminometer (Berthold Technologies).

### Histology and confocal microscopy

Spleens were snap frozen in liquid nitrogen immediately after excision and stored at −80°C. Tissues were subsequently embedded in OCT compound (Sakura Finetek) and 6-μm sections were cut and fixed in acetone at 4°C for 20 min. Sections were rehydrated in TBS and blocked in 10% horse serum in TBS for 1 h at room temperature. Primary Abs (rabbit anti-mouse TTP, H-120, Santa Cruz Biotechnology; sheep anti-mouse IgD, Abcam; rat anti-mouse F4/80, AbD Serotec) or appropriate species isotype controls were incubated overnight at 4°C in blocking buffer. Sections were washed three times in TBS and incubated for 1 h at room temperature with secondary Abs (donkey anti-rabbit IgG–Alexa Fluor 555, donkey anti-sheep IgG–Alexa Fluor 488, donkey anti-rat IgG–Alexa Fluor 647; all Jackson ImmunoResearch Laboratories). Sections were washed three times and mounted in DABCO (2.5% [w/v] diazabicyclo-octane in 90% glycerol/10% PBS [pH 8.6]). Images were captured by confocal microscopy using a Zeiss Axiovert UV microscope with a ×40 objective and processed using an LSM image browser (Zeiss). Paraffin sections were dewaxed with xylene and rehydrated to water through graded alcohol steps. H&E staining was performed using standard methodologies.

### Statistical analysis

GraphPad Prism (version 5.03) was used for statistical analysis. An unpaired, two-tailed Student *t* test was applied for comparison of two groups. For analysis of multiple groups, ANOVA was used with Bonferroni correction for multiple comparisons. A *p* value <0.05 was considered significant.

Probe intensity values for the microarray were analyzed with Partek Genomics Suite version 6.6, build 6.13.0315. A two-way mixed model ANOVA was performed on the entire dataset (55,681 probes) to calculate pairwise contrasts consisting of a corrected step-up *p* value (false discovery rate by the Benjamini–Hochberg step-up method, integrated into the Partek software) and a fold change or ratio for difference in gene expression. The ANOVA was calculated using the following factors: genotype, *Zfp36^+/+^* versus *Zfp36aa/aa*; condition, unstimulated versus 1 h LPS. Weakly expressed genes were filtered out when their sample intensity values (inverse log_2_) did not exceed an arbitrary value of 100 in at least two of three replicate samples from LPS-treated BMMs of either genotype. Probes were also filtered out that contained aberrant outliers (SD of three replicates exceeding average of three replicates). The microarray data described in this study have been submitted to the Gene Expression Ominbus at National Center for Biotechnology Information (GSE68449). (http://www.ncbi.nlm.nih.gov/).

## Results

### Gene targeting to disrupt MK2-mediated phosphorylation of TTP

**FIGURE 1.**
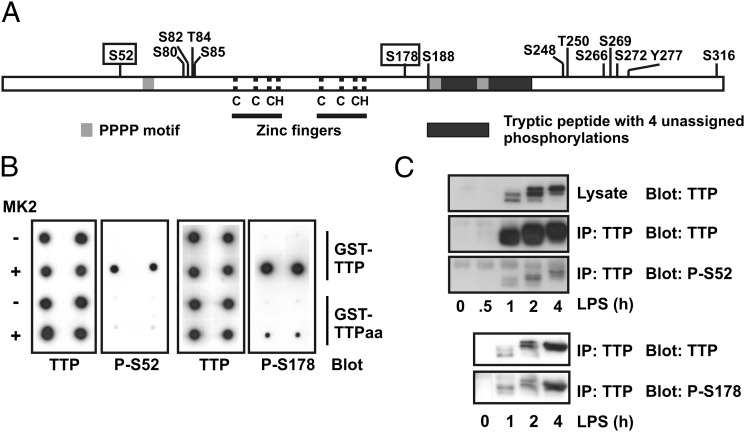
Identification of sites of phosphorylation of TTP in LPS-stimulated myeloid cells. (**A**) Schematic of phosphorylation sites identified in RAW264.7 cells expressing flag-TTP. Serines 52 and 178 are boxed. Pale gray bars indicate tetraprolin (PPPP) motifs. The dark gray bar indicates a large tryptic peptide that contained up to four phosphorylations, only one of which (S188) could be assigned with confidence. Dotted lines indicate position of C_3_H zinc finger motifs. (**B**) Validation of phospho-specific antisera. Phospho-specific antisera were raised against the phosphopeptides LTGRSTpSLVEGR (S52) and LRQSIpSFSGLPSGR (S178). GST-TTP and GST-TTP-S52A/S178A (-TTPaa) were mock treated (−) or phosphorylated in vitro using MK2 (+) as described in *Materials and Methods*, transferred to nitrocellulose, and dot blotted using either a previously described anti-TTP antiserum (20) or the phospho-specific antisera. (**C**) LPS-induced phosphorylation of endogenous murine TTP at serines 52 and 178. RAW264.7 cells were stimulated with 10 ng/ml LPS for the indicated times, lysates were prepared, and endogenous TTP was immunoprecipitated using an antiserum raised against the N terminus of murine TTP (20). Immunoprecipitates were Western blotted using either phospho-specific antisera or an anti-TTP antiserum, with HRP-conjugated protein G for detection. The experiment was performed twice for each phospho-specific antiserum.

Previous studies described extensive phosphoproteomic analysis of exogenous human TTP in HEK293 cells, which express little or no endogenous TTP protein and were not stimulated (24, 25). To identify phosphorylations occurring in a myeloid cell responding to a proinflammatory stimulus, epitope-tagged TTP was immunoprecipitated from a stably transfected and LPS-stimulated RAW264.7 cell line, which expressed exogenous TTP at a level only 2- to 3-fold higher than that of endogenous TTP. Phosphoproteomic analysis unequivocally identified 14 sites of phosphorylation (11 serines, 2 threonines, and 1 tyrosine). Among these were serines 52 and 178 (Fig. 1A). One tryptic peptide, PGPELSPSPTSPTATPTTSSR, was phosphorylated at up to three of the underlined residues. A second large peptide corresponding to residues 188–234 of murine TTP contained up to four discrete phosphorylations that could not be accurately assigned. Broadly, the pattern of phosphorylation was similar to that of human TTP in HEK293 cells (24, 25), although the previous studies did not report phosphorylation of Ser^60^ (which corresponds to Ser^52^ of the murine protein). It was confirmed by in vitro phosphorylation of recombinant wild-type or mutagenized TTP, mass spectrometry, and two dimensional phosphopeptide mapping that MK2 efficiently phosphorylates both Ser^52^ and Ser^178^ (data not shown).

Phospho-specific Abs were raised against Ser^52^ and Ser^178^, and their specificity was established using wild-type or mutant GST-TTP phosphorylated in vitro by MK2 (Fig. 1B). The ability of the Abs to recognize recombinant TTP was dependent on MK2 and intact phospho-acceptor sites. The phospho-specific Abs both recognized endogenous TTP immunoprecipitated from LPS-treated RAW264.7 cells (Fig. 1C), providing further evidence that these sites are phosphorylated in the context of the response to LPS. Incubation of RAW264.7 cells with a p38 inhibitor decreased both Ser^52^ and Ser^178^ phospho-TTP signals (data not shown). As previously reported (17), inhibition of p38 MAPK also decreased levels of total TTP. Therefore, at this stage it cannot be formally concluded that in vivo phosphorylation of S52 and S178 is dependent on p38 MAPK. Evidence in support of this hypothesis is presented below.

**FIGURE 2.**
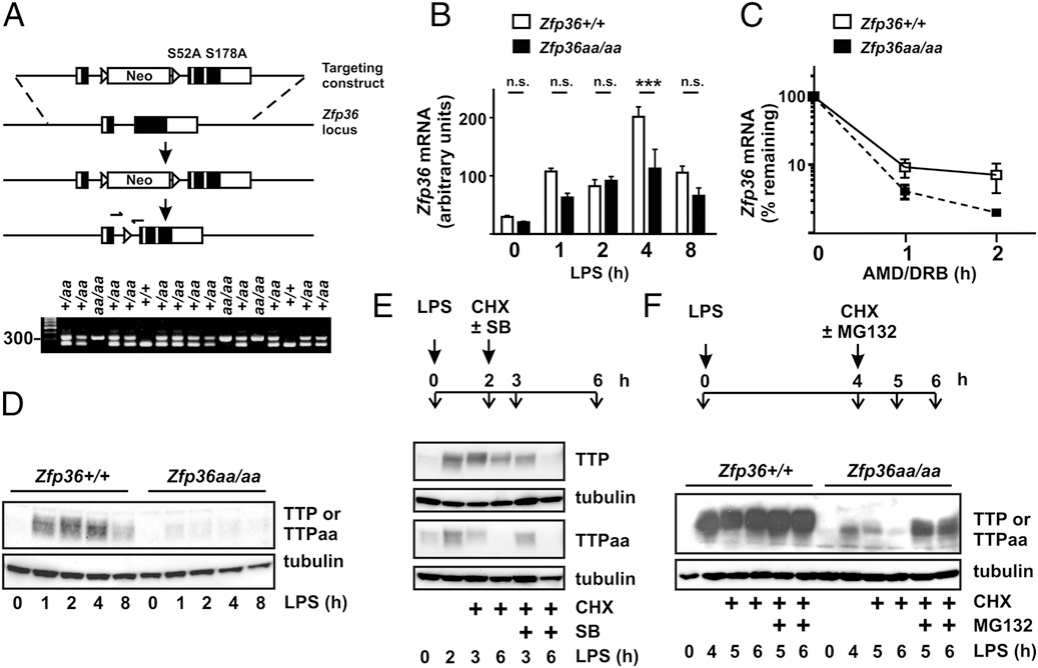
Generation and initial characterization of the *Zfp36aa/aa* strain. (**A**) Schematic of gene targeting strategy. Exons are shown as boxes, Flox sites as arrowheads. The first downward arrow represents homologous recombination of the targeting fragment into the *Zfp36* locus, and the second represents Cre-mediated excision of the neomycin cassette. Left and right arrows represent the primers used in genotyping, that is, CLA1-For and CLA1-Rev. A representative genotyping gel is shown for three litters of mice born from *Zfp36+/aa* crosses. The wild-type locus yields a 234-bp PCR product, the modified locus a 328-bp product. (**B**) *Zfp36^+/+^* and *Zfp36aa/aa* BMMs were treated with 10 ng/ml LPS for 1, 2, 4, and 8 h, and *Zfp36* mRNA was measured by quantitative PCR with normalization first against *Gapdh* then against expression level in *Zfp36^+/+^* cells treated with LPS for 1 h. Means ± SEM of four independent experiments are shown. ***p < 0.005. (**C**) *Zfp36^+/+^* and *Zfp36aa/aa* BMMs were treated with 10 ng/ml LPS for 4 h, actinomycin D (AMD; 5 μg/ml) and DRB (50 μM) were added, and the subsequent decay of *Zfp36* mRNA was monitored by quantitative PCR. Means ± SEM of four independent experiments are shown. (**D**) *Zfp36^+/+^* and *Zfp36aa/aa* BMMs were treated as in (B) and levels of TTP protein were assessed by Western blotting. (**E**) As indicated in the schematic, *Zfp36^+/+^* and *Zfp36aa/aa* BMMs were treated with LPS for 2 h, then 5 μg/ml cycloheximide (CHX) was added in the presence of 5 μM SB202190 (SB) or vehicle control (0.01% DMSO). Cells were harvested at 0, 2, 3, or 6 h as indicated, and TTP was detected by Western blotting. (**F**) As indicated in the schematic, *Zfp36^+/+^* and *Zfp36aa/aa* BMMs were treated with LPS for 4 h, then cycloheximide (CHX) was added in the presence of 5 μg/ml MG132 or vehicle control (0.01% DMSO). Cells were harvested at 0, 4, 5, or 6 h as indicated, and TTP was detected by Western blotting. Western blots in (D)–(F) are representative of at least three independent experiments.

Functional consequences of TTP phosphorylation have so far been studied in vitro or in transfected cells but not in vivo. Therefore, homologous recombination was used to replace both serine 52 and 178 codons with alanine codons in the endogenous murine *Zfp36* locus (Fig. 2A). We refer to the modified locus as *Zfp36aa* and its protein product as TTPaa. In crosses between heterozygous *Zfp36^+^/aa* parents, 105 of 442 (24%) of genotyped pups were *Zfp36aa/aa*. The strain has been backcrossed against the C57BL/6J background for 10 generations. *Zfp36aa/aa* mice are healthy and fertile, with no discernible phenotype under standard conditions of maintenance. Leukocyte populations in spleen, thymus, and peripheral blood were not significantly different from those of wild-type littermates (data not shown).

### TTPaa is weakly expressed

*Zfp36aa* mRNA was expressed at lower levels than *Zfp36* mRNA in BMMs (Fig. 2B), and this was accompanied by a decrease in mRNA stability (Fig. 2C). TTP is thought to autoregulate its expression via binding to adenosine/uridine-rich elements in the *Zfp36* 3′ UTR and destabilization of *Zfp36* mRNA (3, 16, 26). The simplest interpretation of our findings is that the mutant form of TTP has greater mRNA destabilizing activity and enhances the degradation of its own (i.e., *Zfp36aa*) mRNA.

Relative to its wild-type counterpart, TTPaa was weakly expressed in LPS-treated BMMs (Fig. 2D). On the basis of scanning densitometry in four independent experiments, we estimate an 80% decrease in levels. Because this was greater than the decrease in mRNA expression, cycloheximide chases were performed to investigate protein stability (Fig. 2E). Wild-type TTP was relatively stable (*top panel*, *lanes 2–4*), but was rapidly degraded following addition of a p38 MAPK inhibitor (*top panel*, *lanes 5* and *6*). In contrast, TTPaa was rapidly degraded whether the p38 MAPK inhibitor was absent or present (*third panel*, *lanes 2–6*). A proteasome inhibitor protected TTPaa from degradation (Fig. 2F, *last two lanes*). Taken together, these observations confirm that p38 MAPK-dependent phosphorylation of Ser^52^ and Ser^178^ protects TTP protein from degradation by the proteasome (17, 18). Therefore, the diminished expression of TTPaa appears to result from (at least) two distinct phenomena: 1) an increased rate of degradation of *Zfp36aa* mRNA, and 2) an increased rate of degradation of TTPaa protein by the proteasome.

### Selective impairment of responses to LPS in *Zfp36aa/aa* macrophages

**FIGURE 3.**
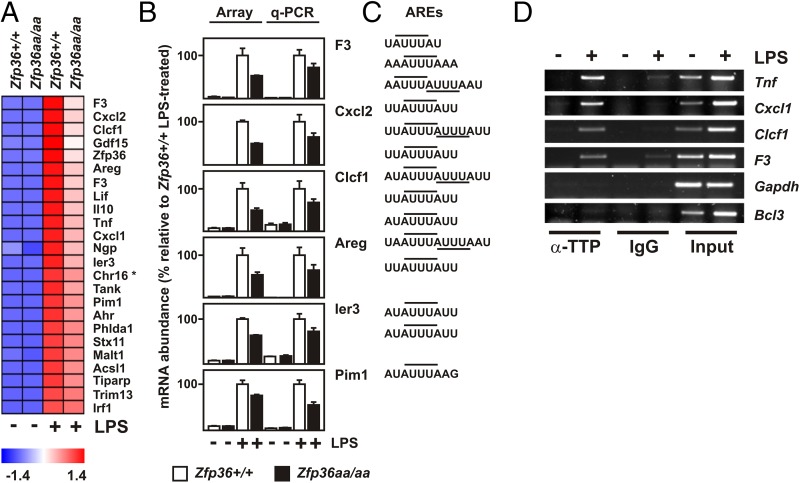
Microarray analysis of differential gene expression in *Zfp36^+/+^* and *Zfp36aa/aa* BMMs. (**A**) *Zfp36^+/+^* and *Zfp36aa/aa* BMMs were stimulated with 10 ng/ml LPS for 1 h, RNA was isolated, and gene expression was analyzed by Agilent microarray. Transcripts upregulated at least 3-fold by LPS and significantly (*p* < 0.05) underexpressed by *Zfp36aa/aa* BMMs are indicated. The heat map represents average normalized intensity values from three independent samples per condition, ranked in order of relative expression level in *Zfp36aa/aa* BMMs (i.e., *F3* being the most strongly underexpressed transcript). Blue represents low and red high relative expression. The *p* values were calculated by two-way ANOVA test using Partek genomics suite. (**B**) *Zfp36^+/+^* and *Zfp36aa/aa* BMMs were stimulated with 10 ng/ml LPS for 1 h and selected transcripts were measured by quantitative PCR. For comparison, microarray-based quantifications are illustrated on the same graphs. In each case, expression levels were normalized against those in LPS-treated *Zfp36^+/+^* BMMs. Means ± SEM of three (microarray) or four (quantitative PCR) independent experiments are indicated. (**C**) Possible TTP binding sites (AUUUA pentamer motifs indicated by horizontal lines) in the 3′ UTRs of selected transcripts. (**D**) *Zfp36^+/+^* BMMs were left untreated or stimulated with 10 ng/ml LPS for 1 h. Immunoprecipitation was carried out using an antiserum against murine TTP or a rabbit IgG control, and the indicated transcripts were detected by RT-PCR. Input RNA from untreated or LPS-stimulated BMMs was also amplified under identical conditions.

Microarray analysis was carried out to identify genome-wide effects of *Zfp36* gene targeting on the LPS responses of macrophages. Of 2130 transcripts significantly induced by 1 h treatment with LPS, *Zfp36aa/aa* BMMs significantly underexpressed 165 (8%) (Table I). The proportion of LPS-induced transcripts that were underexpressed by *Zfp36aa/aa* BMMs remained similar when a 3-, 5-, or 10-fold cut-off for LPS response was applied, or when the stimulus was applied for 4 h rather than 1 h (Table I, Supplemental Table I, GSE68449). Clearly, mutation of the *Zfp36* gene did not globally impair macrophage responses to LPS. Instead, expression of a distinct subset of genes was altered. Fig. 3A illustrates in heat map form 24 transcripts that were induced at least 3-fold by LPS and significantly underexpressed by *Zfp36aa/aa* BMMs. So far, 12 of these differentially expressed transcripts have been validated by quantitative RT-PCR (Figs. 2B, 3B and data not shown; also see Fig. 5B), with good agreement between microarray-based and quantitative RT-PCR–based measurements (Fig. 3B). The list of underexpressed transcripts at 1 h included *Tnf*, *Cxcl1*, *Cxcl2*, and *Il10*, all of which are well-established targets of negative regulation by TTP in myeloid cells (6). *Zfp36* itself was also significantly underexpressed, consistent with the suggestion that TTP autoregulates its own expression. Two alternative *F3* transcripts were underexpressed by *Zfp36aa/aa* BMMs. *F3* encodes tissue factor, an initiator of coagulation that has recently been identified as a target of TTP in macrophages (27). *Ier3*, *Pim1*, and *Lif* were previously identified as TTP targets in cell types other than primary macrophages (28–30). To our knowledge, *Areg* and *Clcf1* have not been identified as targets of TTP; however, their 3′ UTRs have highly conserved adenosine/uridine-rich elements with matches to the consensus TTP binding sequence UAUUUAU (Fig. 3C). As expected, *Tnf* and *Cxcl1* mRNAs were detected in TTP immunoprecipitates from LPS-activated wild-type BMMs (Fig. 3D). *F3* and *Clcf1* mRNAs were also enriched in TTP immunoprecipitates, whereas *Bcl3* mRNA, which lacks a consensus TTP binding sequence, was strongly induced by LPS but did not interact with TTP. As an additional negative control, *Gapdh* mRNA was not enriched in TTP immunoprecipitates. These observations confirm that tissue factor expression is regulated by TTP, and they suggest that *Clcf1* may also be an authentic novel target of TTP. Genome-wide alterations of expression may therefore be a useful starting point for the identification of transcripts regulated by the p38 MAPK/MK2/TTP axis in macrophages or other cell types.

**Table I.** Genome-wide dysregulation of responses to LPS in *Zfp36aa/aa* BMMs

LPS-Induced Transcripts	1 h			4 h		
		↓ in *Zfp36aa/aa*	↑ in *Zfp36aa/aa*		↓ in *Zfp36aa/aa*	↑ in *Zfp36aa/aa*
All	2130	165 (8%)	60 (3%)	4933	291 (6%)	151 (3%)
>3-fold	434	24 (6%)	14 (3%)	1441	103 (7%)	47 (3%)
>5-fold	250	17 (7%)	10 (4%)	897	70 (8%)	29 (3%)
>10-fold	142	14 (10%)	6 (4%)	476	37 (8%)	13 (3%)

A number of cytokine- and chemokine-encoding transcripts were found among those underexpressed by *Zfp36aa/aa* BMMs. To extend this observation a preliminary experiment was carried out using an Ab array to survey secretion of inflammatory mediator proteins by LPS-treated *Zfp36aa/aa* BMMs (Supplemental Fig. 1). Expression of TNF, CCL3, CXCL1, CXCL2, CXCL10, IL-1B, CSF2, and other cytokines or chemokines was lower in *Zfp36aa/aa* BMMs, whereas the expression of CCL2 and TIMP1 was unaltered. Many of these changes in the response to LPS are confirmed and discussed in greater detail below.

**FIGURE 4.**
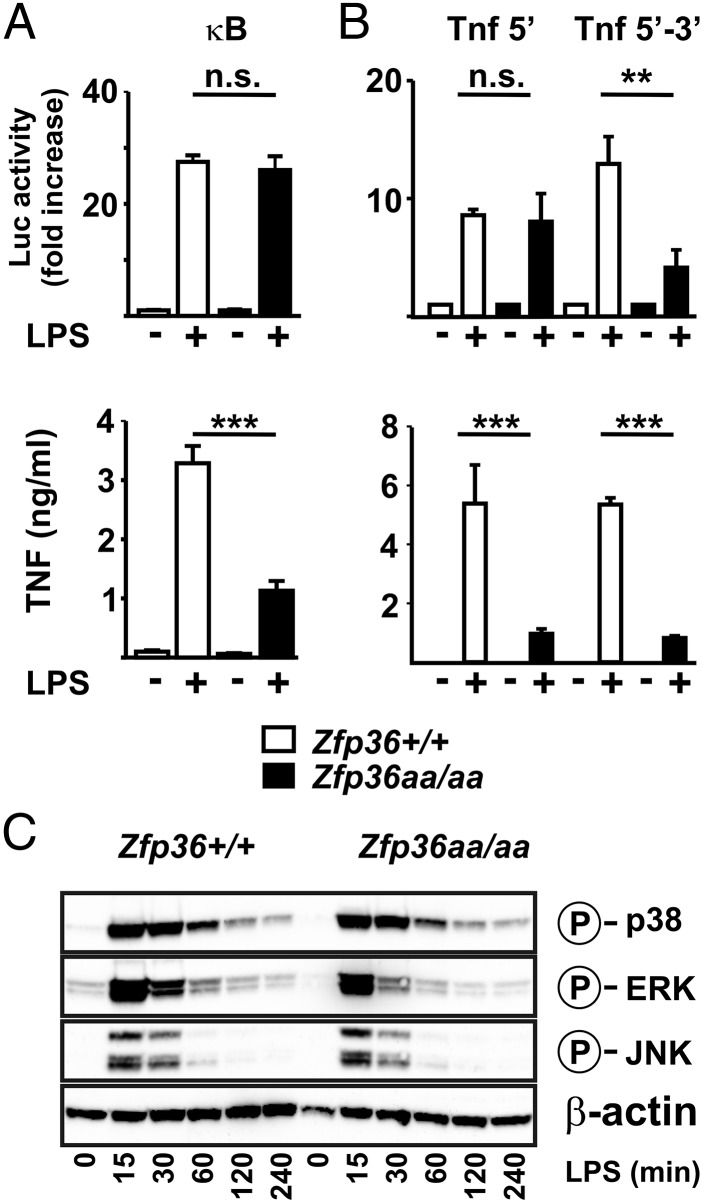
Underexpression of TNF in *Zfp36aa/aa* BMMs does not involve altered transcription, NF-κB signaling, or MAPK signaling. (**A**) *Zfp36^+/+^* or *Zfp36aa/aa* BMMs were transduced with an adenoviral vector expressing firefly luciferase under the control of four tandem consensus NF-κB binding sites. Transduced cells were rested overnight and then stimulated with LPS for 2 h. Luciferase activity was measured (*upper panel*) and secreted TNF protein was measured (*lower panel*). Graphs represent means ± SEM of measurements from three independent BMM cultures of each genotype. (**B**) *Zfp36^+/+^* or *Zfp36aa/aa* BMMs were transduced with adenoviral vectors expressing firefly luciferase under the control of either the *Tnf* 5′ region alone or both the *Tnf* 5′ and 3′ regions. Cells were rested overnight and then stimulated with LPS for 8 h. Luciferase activity was measured (*upper panel*) and secreted TNF protein was measured (*lower panel*). Graphs represent means ± SEM of measurements from three independent BMM cultures of each genotype. (**C**) *Zfp36^+/+^* or *Zfp36aa/aa* BMMs were treated with 10 ng/ml LPS for 0–240 min as indicated, and lysates were prepared and Western blotted using Abs specific for phosphorylated (activated) p38 MAPK, ERK, or JNK. β-Actin was blotted as a loading control. Blots are representative of two independent experiments. *p < 0.05, **p < 0.01, ***p < 0.005.

The effects of TTP mutation on the response to LPS might be explained by alterations in NF-κB or MAPK signaling pathways (28, 31, 32) or otherwise by inhibition of transcription (33). However, LPS-induced activation of an NF-κB reporter was indistinguishable between *Zfp36^+/+^* and *Zfp36aa/aa* BMMs (Fig. 4A, *upper panel*). In the same experiment, TNF protein was underexpressed by *Zfp36aa/aa* BMMs (Fig. 4A, *lower panel*). A reporter construct in which luciferase expression is driven by the TNF promoter (23) was similarly responsive to LPS in *Zfp36aa/aa* and *Zfp36^+/+^* BMMs (Fig. 4B, *upper panel*). In contrast, a reporter containing both the TNF promoter and 3′ UTR was significantly less responsive to LPS in *Zfp36aa/aa* BMMs, suggesting that the inhibitory effect of the TTP mutation depends on the 3′ UTR rather than the promoter of the *Tnf* gene. In the same experiment, endogenous TNF protein was underexpressed by *Zfp36aa/aa* BMMs (Fig. 4B, *lower panel*). As a means of assessing transcription of the endogenous *Tnf* gene, unspliced primary transcript levels were measured in *Zfp36^+/+^* and *Zfp36aa/aa* BMMs. In comparison with wild-type controls, *Zfp36aa/aa* BMMs expressed lower amounts of mature *Tnf* mRNA but unchanged amounts of *Tnf* primary transcript (data not shown). LPS-induced activation of MAPK pathways was also indistinguishable in *Zfp36aa/aa* and *Zfp36^+/+^* BMMs (Fig. 4C). Although targeted mutation of TTP altered the expression of a subset of LPS-induced genes, we found no evidence that it did so by altering NF-κB activation, MAPK signaling, or transcription.

**FIGURE 5.**
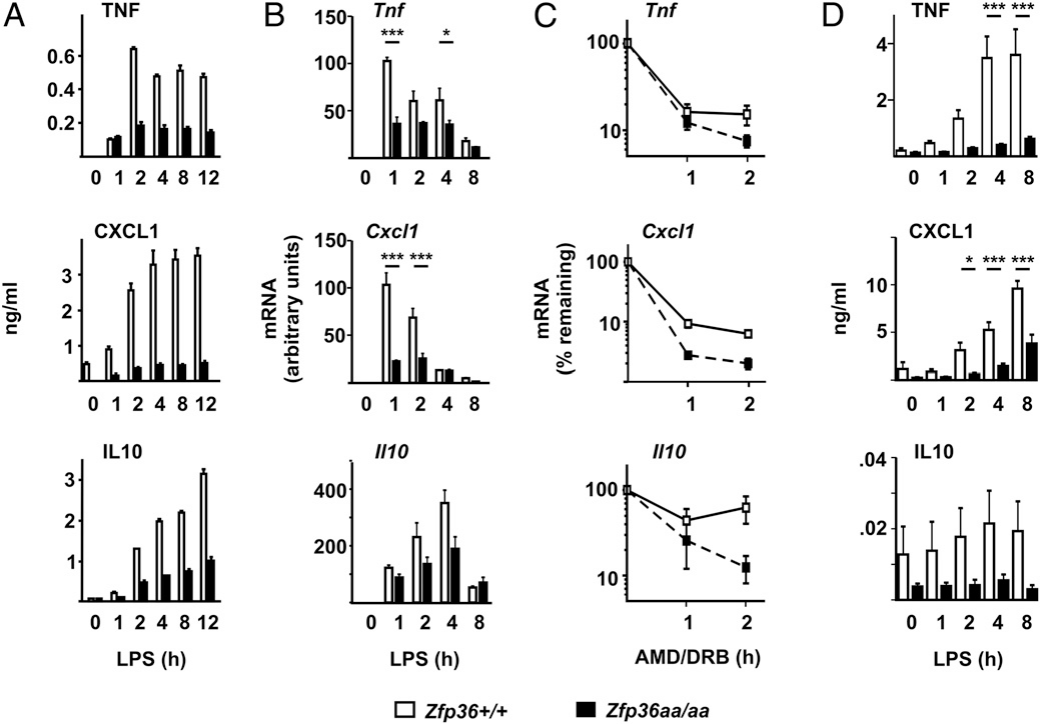
TTPaa decreases expression of target genes via increased mRNA degradation. (**A**) *Zfp36^+/+^* and *Zfp36aa/aa* BMMs were treated with 10 ng/ml LPS for 1, 2, 4, 8, or 12 h, supernatants were harvested, and the indicated factors were measured by ELISA. Means ± SD of triplicate measurements are shown for one representative of four independent experiments. (**B**) *Zfp36^+/+^* or *Zfp36aa/aa* BMMs were treated with 10 ng/ml LPS for 1, 2, 4, or 8 h and the indicated mRNAs were measured by quantitative PCR with normalization to *Gapdh* and against the *Zfp36^+/+^* 1 h time point. Means ± SEM of three independent experiments are shown. (**C**) *Zfp36^+/+^* or *Zfp36aa/aa* BMMs were treated with 10 ng/ml LPS for 1 h and then DRB (50 μM) and actinomycin D (AMD, 5 μg/ml) were added. The indicated mRNAs were measured by quantitative PCR at 0, 1, or 2 h after addition of DRB and AMD and normalized to the 0 h time point within each genotype. Means ± SEM of three to four independent experiments are shown. (**D**) Freshly isolated *Zfp36^+/+^* and *Zfp36aa/aa* peritoneal macrophages were treated with 10 ng/ml LPS for 1, 2, 4, and 8 h, supernatants were harvested, and levels of the indicated cytokines were measured by multiplex bead capture assay. Means ± SEM from four mice of each genotype are shown. *p < 0.05, **p < 0.01, ***p < 0.005.

To further investigate the properties of *Zfp36aa/aa* macrophages, we focused on well-known TTP targets, TNF, CXCL1, and IL-10. All of these proteins were consistently underexpressed by *Zfp36aa/aa* macrophages throughout a 12-h time course, demonstrating that the TTP mutation does not merely delay the response to LPS (Fig. 5A). The corresponding mRNAs were also consistently underexpressed (Fig. 5B), and their half-lives were decreased (Fig. 5C). Accelerated degradation of other known TTP target transcripts such as *F3* and *Cxcl2* was also demonstrated in *Zfp36aa/aa* BMMs (data not shown). To confirm that the underexpression of cytokines or chemokines was not an artifact of the in vitro differentiation of BMMs, peritoneal macrophages of >90% purity were isolated from *Zfp36^+/+^* and *Zfp36aa/aa* mice and stimulated with LPS. Again, there was consistent underexpression of TNF and CXCL1 by *Zfp36aa/aa* macrophages (Fig. 5D). Expression of IL-10 was very low, and neither the response to LPS nor the difference between *Zfp36^+/+^* and *Zfp36aa/aa* macrophages achieved statistical significance. These experiments confirm that although *Zfp36aa/aa* macrophages express low levels of TTP protein, this mutant form of TTP is highly active and promotes destabilization of target mRNAs, many of which are involved in the inflammatory response to TLR4 engagement.

### *Zfp36aa/aa* mice display decreased systemic responses to LPS

**FIGURE 6.**
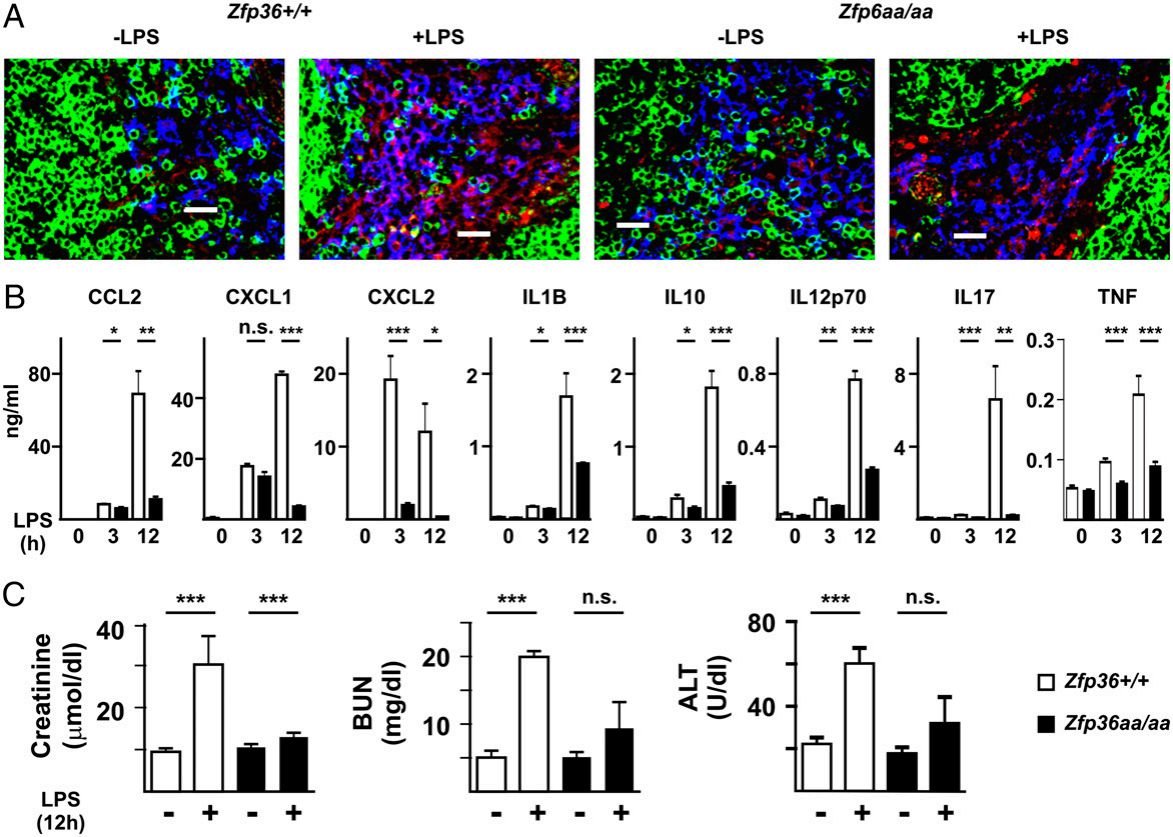
*Zfp36aa/aa* mice are protected from experimental endotoxemia. (**A**) *Zfp36^+/+^* and *Zfp36aa/aa* mice were injected i.p. with LPS (5 mg/kg) for 12 h and expression of TTP was assessed by confocal imaging of spleen cryosections stained with anti-IgD (green), anti-TTP (red), or F4/80 (blue, original objective lens magnification ×40). Scale bars, 20 μm. Images are representative of four individual mice per group. (**B**) *Zfp36^+/+^* and *Zfp36aa/aa* mice were injected i.p. with LPS (5 mg/kg). After 3 or 12 h mice were exsanguinated and serum levels of cytokines were measured by multiplex bead capture assay. Graphs show mean ± SEM cytokine concentrations from 10 mice of each genotype at 3 and 12 h, and five untreated mice. (**C**) Serum levels of tissue damage markers were measured at the 12 h time point in the same sera as in (B). *p < 0.05, **p < 0.01, ***p < 0.005.

Tissue-specific deletion of the *Zfp36* gene in myeloid cells rendered mice extremely sensitive to LPS-induced endotoxemia (3, 13). It was hypothesized that a gain of function mutation of TTP would have the opposite effect. Systemic challenge of *Zfp36^+/+^* mice by i.p. injection of LPS increased the expression of TTP protein in spleen (Fig. 6A). TTP staining was principally cytoplasmic as expected, weak in the B and T cells of the white pulp, most evident in F4/80^+^ red pulp macrophages, but also detectable in some F4/80^−^ cells. Although this method is not highly quantitative, expression of TTPaa protein appeared to be weaker than that of the wild-type protein (Fig 6A, compare *second* and *fourth panels*). Serum levels of CCL2, CXCL2, IL-1B, IL-10, IL-12p70, IL-17, and TNF were elevated in response to LPS and present at significantly lower levels in serum of *Zfp36aa/aa* mice both 3 and 12 h after injection of LPS (Fig. 6B). CXCL1 was significantly underexpressed by *Zfp36aa/aa* mice at the 12 h time point. Underexpression of CXCL1 by *Zfp36aa/aa* mice at the 3 h time point was not statistically significant in the experiments shown, but it was so in other, similar experiments (see figures 8D and 5D of the accompanying manuscript, that is, Ref. 34). Creatinine and blood urea nitrogen (serum markers of renal damage) and alanine transaminase (a marker of hepatic damage) were elevated in *Zfp36^+/+^* mice 12 h after injection of LPS (Fig. 6C). In *Zfp36aa/aa* mice, these markers of LPS-induced organ damage were increased only marginally or not significantly.

### Acquired immune responses to *S. typhimurium* are not strongly impaired in *Zfp36aa/aa* mice

The mutation of two serine codons effectively increased TTP activity by preventing its inactivation, impaired the expression of several inflammatory mediators, and conferred significant protection in an experimental model of endotoxemia. However, these codons are subject to strong positive selective pressure, apparently being conserved throughout vertebrate evolution (25). It was hypothesized that the loss of phosphorylation-mediated dynamic control of TTP function may impair adaptive immune responses, causing increased susceptibility to pathogens. By way of precedent, disruption of the *Mk2* gene (encoding the kinase that phosphorylates serines 52 and 178 of TTP) increased susceptibility to the intracellular pathogen *Listeria monocytogenes* (35). To test the hypothesis, *Zfp36^+/+^* and *Zfp36aa/aa* mice were inoculated with an attenuated strain of *S. typhimurium*. In this well-established model of infectious disease (22, 36), bacteria typically peak in numbers in the liver and spleen ∼7 d after inoculation, with most being cleared by day 55. Clearance of this strain of *Salmonella* is mediated by a Th1 adaptive immune response and is highly dependent on IFN-γ, but not TNF (22, 37).

**FIGURE 7.**
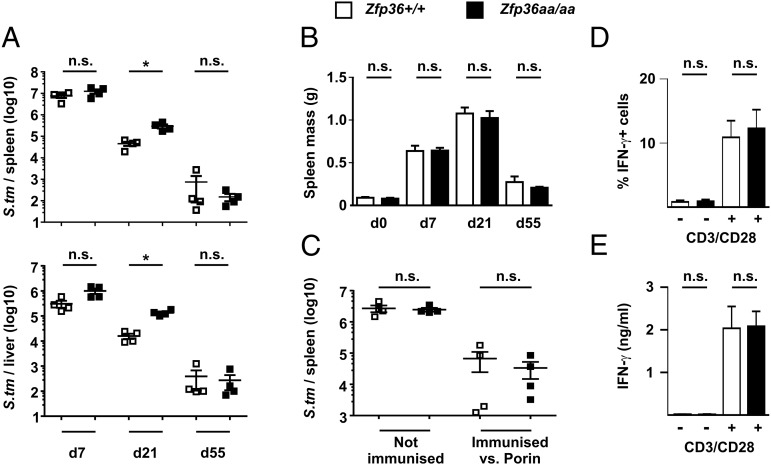
*Zfp36aa/aa* mice retain effective acquired immune responses to *S. typhimurium*. (**A**) *Zfp36^+/+^* and *Zfp36aa/aa* mice were injected i.p. with 5 × 10^5^ *S. typhimurium.* Mice were sacrificied at days 7, 21, or 55, spleens and livers were dissected out, and bacterial burdens in these organs were measured. Graphs represent data from four mice of each genotype. (**B**) Spleens were weighed in control, noninoculated mice or at days 7, 21, or 55 after inoculation with *S. typhimurium*. The graph represents data from four mice of each genotype. (**C**) Four mice of each genotype were either immunized twice with purified porin protein or mock-immunized with PBS and then inoculated with 5 × 10^5^ *S. typhimurium*. Bacterial loads in spleen were measured at day 7. (**D**) Whole splenocytes were stimulated for 6 h using plate-bound anti-CD3 and anti-CD28, and intracellular expression of IFN-γ in CD3^+^CD4^+^ T cells was assessed by flow cytometry. Graph shows mean ± SEM from four mice of each genotype. (**E**) Lymph node T cells were isolated from *Zfp36^+/+^* or *Zfp36aa/aa* mice, stimulated for 72 h using plate-bound anti-CD3 and anti-CD28, and secreted IFN-γ was measured. Graph shows mean ± SEM from three mice of each genotype. Representative of three independent experiments. *p < 0.05, **p < 0.01, ***p < 0.005.

Bacterial loads were significantly higher in spleens and livers of *Zfp36aa/aa* mice 21 d after inoculation, but they were comparable to those in wild-type mice by day 55 (Fig. 7A). Pathogen-induced expansion and subsequent contraction of the spleen followed identical time courses in *Zfp36^+/+^* and *Zfp36aa/aa* mice (Fig. 7B). *Zfp36^+/+^* and *Zfp36aa/aa* mice were vaccinated twice with PBS or purified *S. typhimurium* porin proteins prior to challenge with live *S. typhimurium*. The immunization gave similarly strong (>97.5%) protection against the establishment of bacterial infection in both strains of mice (Fig. 7C).

IFN-γ is reported to be a target of negative regulation by TTP (38). The ability of *Zfp36aa/aa* mice to clear an *S. typhimurium* infection was therefore surprising and prompted us to investigate the effect of TTP mutation on the expression of IFN-γ by T cells. Stimulation of *Zfp36^+/+^* and *Zfp36aa/aa* splenocytes via CD3 and CD28 induced similar numbers of IFN-γ–expressing cells and similar secretion of IFN-γ protein (Fig. 7D, 7E). In summary, we have not yet found evidence of impaired adaptive immune responses in the *Zfp36aa/aa* mouse, consistent with its ability to both clear an *S. typhimurium* infection and mount an effective response to vaccination against this pathogen.

### TTPaa functions as a dominant inhibitor of inflammatory gene expression

**FIGURE 8.**
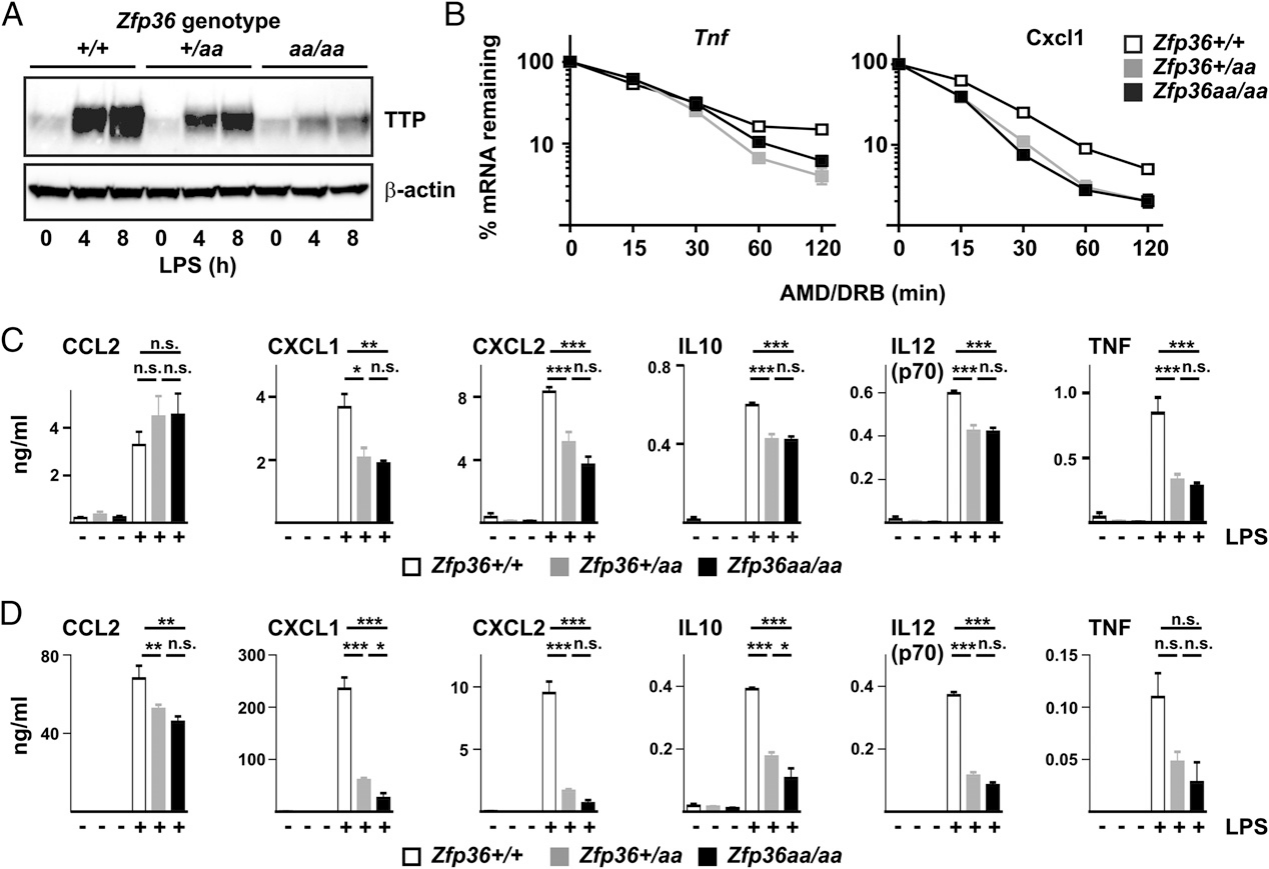
TTPaa is a dominant inhibitor of inflammatory gene expression. (**A**) *Zfp36^+/+^*, *Zfp36^+^/aa*, and *Zfp36aa/aa* BMMs were treated with 10 ng/ml LPS for 0, 4, or 8 h and expression of TTP protein was determined by Western blotting. β-Actin was blotted as a loading control. Results are representative of four similar experiments. (**B**) *Zfp36^+/+^*, *Zfp36^+^/aa*, and *Zfp36aa/aa* BMMs were treated with 10 ng/ml LPS for 1 h and then DRB (50 μM) and actinomycin D (AMD, 5 μg/ml) were added. The indicated mRNAs were measured by quantitative PCR at 0, 15, 30, 60, or 120 min after addition of DRB and AMD and normalized to the 0 h time point within each genotype. Means ± SEM of three to four independent experiments are shown. Error bars are obscured by the symbols. (**C**) *Zfp36^+/+^*, *Zfp36^+^/aa*,and *Zfp36aa/aa* BMMs were treated with 10 ng/ml LPS for 4 h and levels of the indicated cytokines in the supernatants were measured by multiplex bead capture assay. Means ± SEM from four mice of each genotype are shown. (**D**) *Zfp36^+/+^*, *Zfp36^+^/aa*, and *Zfp36aa/aa* mice were injected i.p. with LPS (5 mg/kg). After 3 h mice were humanely culled, exsanguinated, and serum levels of cytokines were measured by multiplex bead capture assay. Graphs show means ± SEM cytokine concentrations from at least four mice of each genotype. *p < 0.05, **p < 0.01, ***p < 0.005.

Substitution of two phospho-acceptor sites of TTP by nonphosphorylatable residues appeared to cause a gain of function, increasing the rate of degradation of target mRNAs and impairing the expression of inflammatory mediators. To test whether the mutant allele exerts a dominant phenotype in the presence of a wild-type allele, *Zfp36^+/+^* and *Zfp36aa/aa* mice were bred to generate heterozygotes. All of these mice are derived from the same breeding program and have as near as possible the same genetic background with the exception of the *Zfp36* locus. As previously shown, the expression of TTP protein was high in *Zfp36^+/+^* BMMs and comparatively low in *Zfp36aa/aa* BMMs (Fig. 8A). In heterozygous BMMs, the levels of TTP were intermediate, with an estimated 10–20% of this being the mutant form of TTP. Rates of degradation of *Tnf* and *Cxcl1* mRNA were similar in *Zfp36+/aa* and *Zfp36aa/aa* BMMs, and greater than in *Zfp36^+/+^* BMMs (Fig. 8B). CXCL1, CXCL2, IL-10, IL-12p70, and TNF were all significantly underexpressed by both *Zfp36+/aa* and *Zfp36aa/aa* BMMs, with no significant difference between these genotypes (Fig. 8C). The LPS-induced expression of CCL2 did not differ significantly between *Zfp36^+/+^*, *Zfp36^+^/aa*, and *Zfp36aa/aa* BMMs, confirming that the targeted mutation of TTP phospho-acceptor sites does not globally impair the response to engagement of TLR4. After i.p. injection of LPS, serum levels of CXCL1, CXCL2, IL-10, and IL-12p70 were significantly lower in both *Zfp36aa/aa* and *Zfp36^+^/aa* than in *Zfp36^+/+^* control mice (Fig. 8D). In the cases of CXCL1 and IL-10, there were small but statistically significant differences in expression between *Zfp36aa/aa* and *Zfp36^+^/aa* mice. There was a small but statistically significant reduction in the levels of CCL2 in serum of LPS-treated *Zfp36aa/aa* and *Zfp36^+^/aa* mice. TTPaa therefore appears to function as a dominant mRNA destabilizing factor and inhibitor of inflammatory gene expression, even when in competition with a large excess of wild-type protein.

## Discussion

The substitution of only two codons of the endogenous murine locus encoding TTP gave rise to a strong and dominant hypoinflammatory phenotype that is (as far as we are aware) unprecedented for such a minimal genetic modification. The expression of several inflammatory mediators was impaired in LPS-treated peritoneal or bone marrow–derived *Zfp36aa/aa* macrophages. Organ damage and systemic expression of inflammatory mediators in response to LPS were likewise diminished in *Zfp36aa/aa* mice. In some cases (e.g., CXCL2, IL-17), serum levels were >10-fold lower in *Zfp36aa/aa* mice than in wild-type controls. We also found that the genetically modified mice were protected in other models of inflammatory pathology (E.A. Ross, T. Smallie, C.D. Buckley, J.L. Dean, and A.R. Clark, manuscripts in preparation). Note that TTP has been identified as a putative tumor suppressor, which limits the expression of many regulators of cell cycle progression and survival. Loss of expression or phosphorylation-mediated inactivation of TTP have been linked to poor prognosis in several types of cancer (39–41). This raises the intriguing prospect, now under investigation, that the *Zfp36aa/aa* mouse may have a degree of protection in some experimental models of cancer.

It has been suggested that TTP decreases expression of inflammatory mediators in part by impairing the activation of NF-κB (31, 42, 43). Although we did not directly test this hypothesis, our observations suggest that the enhanced anti-inflammatory function of TTPaa is related to the canonical mechanism of mRNA destabilization rather than involving inhibition of NF-κB signaling. Accordingly, several known mRNA targets of TTP were degraded at increased rates in *Zfp36aa/aa* cells, whereas there was no evidence of impaired NF-κB function or decreased transcription of *Tnf*, a well-characterized NF-κB–dependent gene. The phenotype reflects the inability of TTPaa to be inactivated via p38 MAPK–MK2 signaling. TTP has also been reported to impair translation of target transcripts, and phosphorylation of Ser^52^ and Ser^178^ to alleviate this effect (13, 15). In our hands, decreases in expression of cytokines and chemokines by *Zfp36aa/aa* BMMs were generally accompanied by decreases in the stability and the steady-state levels of the corresponding mRNAs. However, it cannot be ruled out that decreased mRNA translation also contributes to the underexpression of inflammatory mediators by *Zfp36aa/aa* cells or animals. If this proves to be correct, experiments such as microarrays, based on quantification of steady-state mRNA, may underestimate the impact of the *Zfp36* mutation.

Expression of TTPaa was ∼5-fold lower than that of the wild-type protein. At least two distinct phenomena contributed to this underexpression. First, the stability of *Zfp36* mRNA was decreased in cells expressing the mutated form of TTP, supporting previous suggestions that TTP autoregulates its expression via destabilization of its own mRNA (3, 16, 26). Second, TTPaa was constitutively unstable, whereas wild-type TTP protein was stabilized in a p38 MAPK-dependent manner. This confirms that p38 MAPK-dependent phosphorylation of TTP at Ser^52^ and Ser^178^ protects it from proteasomal degradation (17, 18). Despite this decrease of expression, the phenotype represents a gain of function, as demonstrated most clearly by the behavior of heterozygous (*Zfp36^+^/aa*) mice or macrophages derived from them. Their expression of inflammatory mediators was as low, or almost as low, as in *Zfp36aa/aa* mice or macrophages. Similar half-lives of target mRNAs and levels of expression of inflammatory mediators in *Zfp36^+^/aa* and *Zfp36aa/aa* macrophages imply that TTPaa effectively competes with an excess of wild-type TTP to promote more rapid mRNA degradation. One possible explanation is that the phosphorylation of Ser^52^ and Ser^178^ decreases the affinity of TTP for RNA, so that the nonphosphorylatable mutant has a competitive advantage in binding to target sequences. The difference in expression of both TTPaa protein and its mRNA targets makes it extremely difficult to test this hypothesis directly in *Zfp36aa/aa* macrophages. However, other researchers have used biochemical methods to demonstrate MK2-mediated decreases in mRNA binding affinity of TTP (44, 45).

The results described in the present study emphasize that the equilibrium between phosphorylated and unphosphorylated TTP (with respect to serines 52 and 178) plays a crucial role in determining the inflammatory output of macrophages. The heterozygous phenotype suggests that quite a small shift in favor of the dephosphorylated form could have strong anti-inflammatory consequences. It follows that endogenous factors or therapeutic reagents could exert anti-inflammatory effects by influencing the phosphorylation/dephosphorylation equilibrium. As an example of the former, an accompanying paper shows that dual specificity phosphatase 1 limits inflammatory responses in vitro and in vivo partly by modulating the phosphorylation status of TTP (34).

There are at least four possible methods for therapeutic targeting of the equilibrium: 1) Inhibitors of p38 MAPK destabilize inflammatory mediator mRNAs by activating TTP (46, 47). However, despite clear therapeutic effects in experimental models of acute and chronic inflammation, these compounds do not have sustained clinical benefits in humans (48). A possible explanation for their transient anti-inflammatory action is that in a chronic setting they prevent the accumulation of TTP protein and therefore have little or no effect on target mRNA stability (46, 49). 2) Disruption of the gene encoding MK2 impairs inflammatory responses of macrophages, identifying MK2 as a candidate target for novel anti-inflammatory drugs (15). However, there are significant technical challenges to the development of MK2 inhibitors (50). 3) It may be possible to increase TTP activity and downregulate expression of inflammatory mediators via agonism of PP2A. Increasing PP2A activity is already under investigation as a therapeutic approach in cancer and neurodegenerative disease (51). 4) In principle, protection of unphosphorylated TTP from proteasome-mediated degradation should shift the balance in favor of degradation of proinflammatory mRNAs. A recent publication describes ubiquitin-independent degradation of TTP as a consequence of the recognition of unstructured protein domains by the proteasome (52). This may provide a starting point for attempts to block the degradation of TTP protein.

Any discussion of therapeutic targeting of TTP needs to be tempered with some caution. Serines 52 and 178 and surrounding residues are conserved throughout vertebrates, implying that MK2-mediated phosphorylation of TTP is an evolutionarily ancient mechanism, subject to strong selective pressure. We hypothesized that *Zfp36aa/aa* mice may have a defect in the acquired immune response to infection. To test this hypothesis we used an attenuated strain of *Salmonella*, which gives rise to a well-characterized, Th1-dominated response (22, 36, 37). The infection is normally cleared within ∼50–60 d, in a manner that depends on IFN-γ but appears independent of TNF. We found only a slight delay in clearance of the bacterium by *Zfp36aa/aa* mice. Total IgG Ab titers against the immunodominant Ag porin were unaltered, although there was some evidence of subtle alterations of Ig class switching, which require further investigation (data not shown). Vaccination using purified porin protein also generated a protective immune response in *Zfp36aa/aa* mice. The behavior of *Zfp36aa/aa* T cells has not yet been investigated in detail, but their expression of IFN-γ was shown to be unimpaired. Clearly, the development of an acquired immune response depends on some functions of innate immune cells, for example Ag presentation. Until such functions have been analyzed in detail we can only conclude that both innate and adaptive immune functions are sufficient for clearance of a model pathogen in *Zfp36aa/aa* mice. This suggests that targeting the TTP phosphorylation/dephosphorylation equilibrium may incur relatively low costs in terms of response to infection. However, the subtle adaptive immune phenotype described in the present study might confer a significant decrease of fitness in the wild, for example in the face of challenge with more virulent pathogens, or there may be some other selective disadvantage that has not yet come to light.

In conclusion, the results described in the present study provide strong evidence that the equilibrium between phosphorylated and unphosphorylated TTP (with respect to Ser^52^ and Ser^178^) is a critical determinant of the strength of inflammatory responses in vitro and in vivo. They also provide proof of the concept that this equilibrium might be targeted as a means of treatment of inflammatory pathology, which may escape some of the problems that have been associated with direct targeting of p38 MAPK itself. Finally, they provide unambiguous corroboration of a working model of TTP expression and function, which to this point was based largely on in vitro assays and overexpression of TTP in transfected cells.

## Supplementary Material

Data Supplement
