# Supplementary material for: Dominant Suppression of Inflammation via Targeted Mutation of the mRNA Destabilizing Protein Tristetraprolin
Source: J Immunol. 2015 May 22;195(1):265–76. doi: 10.4049/jimmunol.1402826 (PMC4472942; doi:10.4049/jimmunol.1402826)
Supplement: Data Supplement [file JI_1402826.zip › JI_1402826_Supplemental_Figure_1.pdf]

A

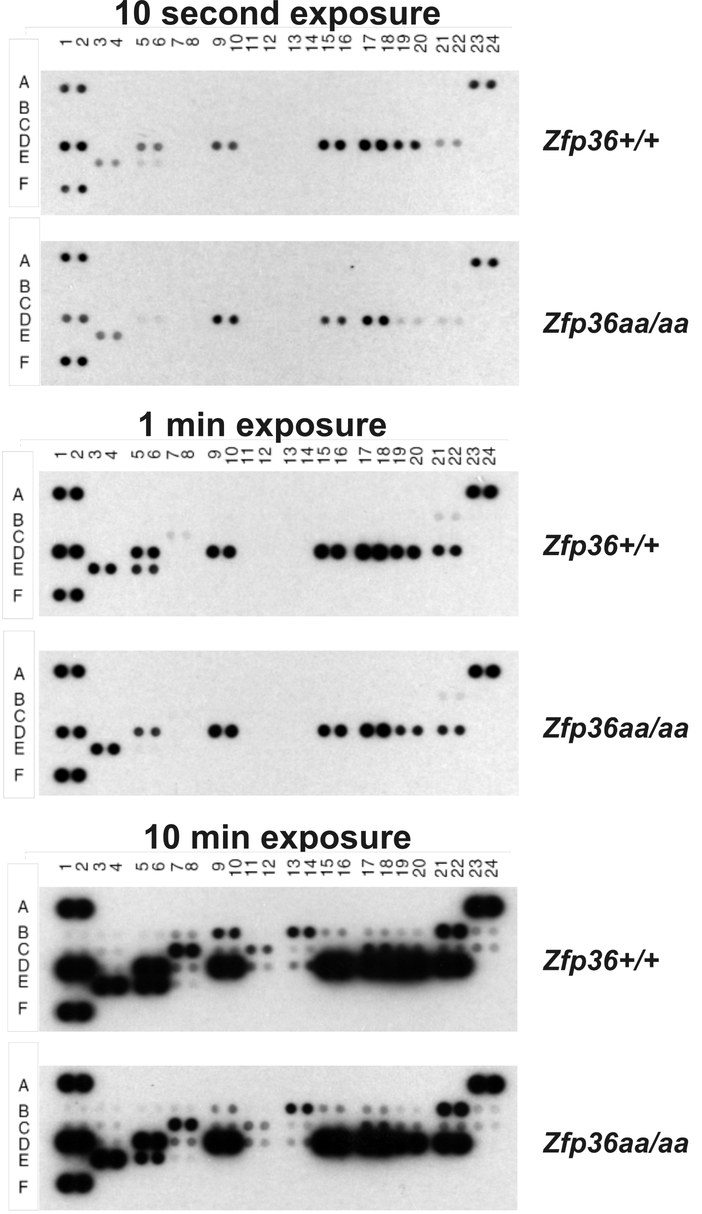

B

Coordinate	Target/Control
A1,A2	Reference spot
A23,A24	Reference spot
B1,B2	CXCL13 (BLC, BCA-1)
B3,B4	C5 (C5a)
B5,B6	CSF3 (G-CSF)
B7,B8	CSF2 (GM-CSF)
B9,B10	CCL1 (TCA-3, I-309)
B11,B12	CCL11 (Eotaxin)
B13,B14	CD54 (sICAM-1)
B15,B16	IFNG (IFN-γ)
B17,B18	IL1A (IL-1α, IL-1F1)
B19,20	IL1B (IL-1β, IL-1F2)
B21,22	IL1RN (IL-1Ra, IL-1F3)
B23,24	IL-2
C1,C2	IL-3
C3,C4	IL-4
C5,C6	IL-5
C7,C8	IL-6
C9,C10	IL-7
C11,C12	IL-10
C13,C14	IL-13
C15,C16	IL-12p70
C17,C18	IL-16
C19,C20	IL-17
C21,C22	IL-23
C23,C24	IL-27
D1,D2	CXCL10 (IP-10, CRG-2)
D3,D4	CXCL11 (I-TAC)
D5,D6	CXCL1 (KC)
D7,D8	CSF1 (M-CSF)
D9,D10	CCL2 (MCP-1, JE)
D11,D12	CCL12 (MCP-5)
D13,D14	CXCL9 (MIG)
D15,D16	CCL3 (MIP-1α)
D17,D18	CCL4 (MIP-1β)
D19,D20	CXCL2 (MIP-2)
D21,D22	CCL5 (RANTES)
D23,D24	CXCL12 (SDF-1)
E1,E2	CCL17 (TARC)
E3,E4	TIMP-1
E5,E6	TNF (TNFα)
E7,E8	TREM-1 (CD354)
F1,F2	Reference spot
F23,F24	PBS (negative control)

C

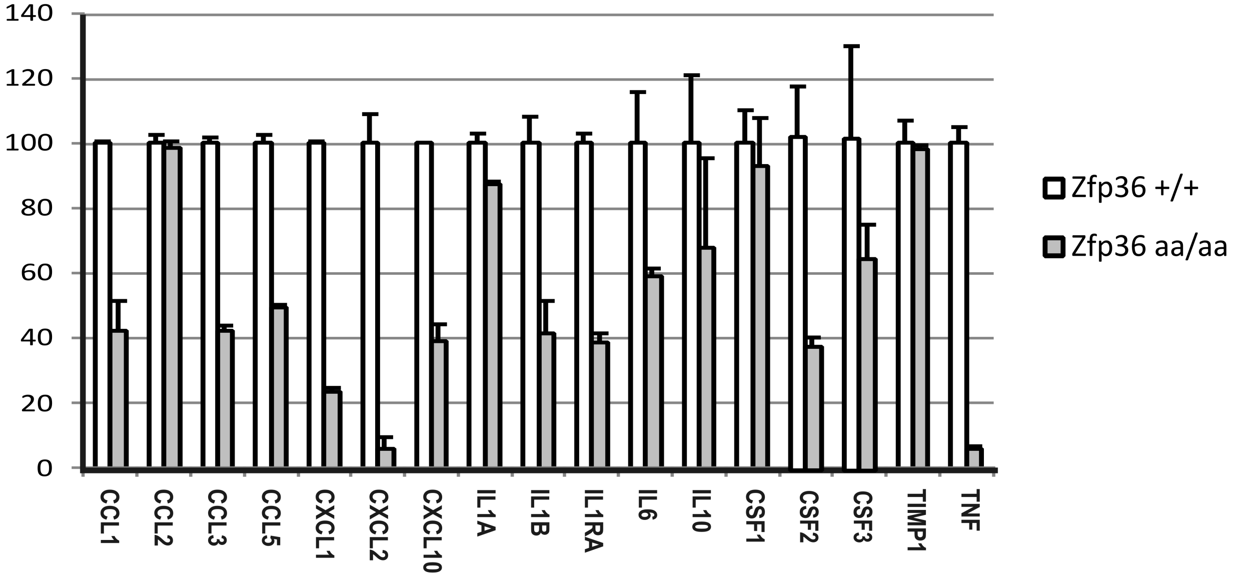

Supplemental Figure 1. Comparison of protein secretion by*Zfp36*+/+ and *Zfp36aa/aa* BMMs. BMMs were stimulated for 4 h with 10 ng/ml LPS, supernatants were harvested and applied to an inflammatory protein expression array according to manufacturer's instructions. **A**) Three different exposures of the two filters. **B**) Key of capture antibody disposition on the array. The linear range of detection was estimated from several different exposures, and relative concentrations of secreted factors were calculated by scanning densitometry for those factors that were detectable within linear range. Some factors could not be assessed because their levels were above or below the linear detection range, or because of strong signals from adjacent spots. **C**) Relative levels of expression in *Zfp36*+/+ and *Zfp36-/-* BMMs. Error bars indicate range of values from duplicate spots.
